# The Value of Acetyl-Salicylic Acid in Certain Affections of the Eye

**Published:** 1910-11-19

**Authors:** John Allan


### THE GENERAL PRACTITIONER'S COLUMN.

[Contributions to this Column are invited, and, if accepted, will be paid for.]

### THE VALUE OF ACETYL-SALICYLIC ACID IN CERTAIN AFFECTIONS OF THE EYE.

By JOHN ALLAN, M.D.

During the last twenty or thirty years many new drugs have been placed on the market. Some of these have enjoyed a brief but passing popularity—a few have stood the test of scientific investigation. Acetyl-salicylic acid is one which merits a place in the medical man's therapeutic armamentarium, and there is good reason to believe that its worth will be recognised by its being included as an official preparation in the next British Pharmacopœia.

In a former communication * I drew attention to the value of this drug in chorea, and now I should like to point out that it may be prescribed with benefit in certain eye diseases. Being one of the "salicylate" group the drug might be expected to prove of greatest service in rheumatic conditions, and, as a matter of fact, experience confirms this. The utility of the drug is, however, by no means limited to this one group of cases.

Let me first of all testify to its value in iritis, and perhaps I can best do so by quoting an illustrative case. P—— O——, a married man, thirty-seven years of age, came under treatment for iritis which was, in the patient's opinion, due to a chill. There was no past or present rheumatic history. Syphilis was denied, and there was not the slightest evidence to suggest it. Other ætiological factors were also negative. The usual subjective symptoms, pain in the eye, photophobia, lachrymation, etc., were present, and objectively the iris had a muddy appearance and pupillary dilation under atropine was poor and irregular. First of all the treatment consisted of frequent bathing with boracic lotion, instillation of atropine drops (8 grains to the ounce), and the administration of an alterative mixture, but no progress was made. The patient was then put to bed and acetyl-salicylic acid, 15 grains, four hourly, was ordered. The response to this treatment was most gratifying, and in three days all the acute symptoms had disappeared. The acetyl-salicylic acid was then reduced to 10 grains thrice daily and continued in these doses for a fortnight, by which time the attack had been completely recovered from.

In making use of this drug in iritis it is most important to treat the patient in bed for a few days and to give large doses. In some cases I have given larger doses than those mentioned above. It may be urged that salicylate of sodium would have acted just as well, but acetyl-salicylic acid is so much more pleasant to take that I think it ought to be prescribed in preference to the other drug. Moreover, it is as a rule much more easily tolerated by patients.

Another affection in which this drug can be beneficially employed is acute conjunctivitis, more particularly in the non-purulent varieties. Again, this drug appears to have a remarkably sedative action in many painful affections of the eye. The pain in the diseases already mentioned is quickly relieved, while the pain which accompanies various injuries to the eye, or pain subsequent to operations on the eye, is soothed by the administration of a large dose of this drug. It has been confidently maintained by Continental investigators that acetyl-salicylic acid has hypnotic properties, and from my personal observation I can state that there seems to be much truth in this assertion. I have time and again afforded patients relief and permitted them to obtain a good night's rest by giving 30 grains of acetyl-salicylic acid at bedtime.

The drug can be given in powder, in cachets, or in a mixture in suspension, and it is of little importance which of these methods is used for its administration. It should not be given in the form of tabloids or tablets.

* The Hospital, July 27, 1907.

